# Comprehensive analysis of root canal morphology in maxillary premolars among the Pakistani subpopulation: a CBCT-based study

**DOI:** 10.1186/s40001-024-01990-6

**Published:** 2024-07-27

**Authors:** Hmoud Ali Algarni, Meshal Aber Alonazi, Hamza Arshad, Fatima Zahra, Fahad Umer, Irfan Maqbool, Azhar Iqbal, Mohmed Isaqali Karobari

**Affiliations:** 1 Department of Restorative Dentistry, College of Dentistry, Jouf University, 72345 Sakaka, Saudi Arabia; 2 Prosthodontics, Dental Section, Department of Surgery, The Aga Khan University, Karachi, Pakistan; 3 Operative Dentistry and Endodontics, Dental Section, Department of Surgery, The Aga Khan University, Karachi, Pakistan; 4 Private Dental Practitioner, Islamabad, Pakistan; 5 Department of Dental Research, CGHR, Saveetha Medical College and Hospitals, Saveetha Institute of Medical and Technical Sciences, Saveetha University, Chennai, 602105 Tamil Nadu India; 6 Department of Restorative Dentistry & Endodontics, Faculty of Dentistry, University of Puthisastra, Phnom Penh, 12211 Cambodia

**Keywords:** Cone beam computed tomography, Dental anatomy, Dental diagnostic imaging, Dental pulp, Endodontics, Morphology, Root, Root canal, Premolars, Pakistani subpopulations

## Abstract

**Background:**

Understanding the root canal morphology is essential for the success of root canal treatment. Therefore, this study aimed to evaluate and analyze the root canal configuration of maxillary premolars using Cone Beam Computed Tomography in the Pakistani subpopulation.

**Method:**

This cross-sectional study utilized CBCT scans from two distinct centres: Aga Khan University in Karachi and Jinnah MRI and Body Scans in Lahore. The CBCT images were visualized using GALAXIS version 1.9 (SICAT GmbH and Co. KG, Bonn, Germany), integrated within the Sirona Dental System (D-64625 Bensheim, Germany). The scanning parameters were standardized at 85 kV, 7 mA, with a 15-s exposure time and a voxel size of 0.16 mm. A total of 707 CBCT scans were collected, encompassing 2180 maxillary premolars. Root canal configurations were classified based on (Ahmed et al. Int Endod J. 2017;50(8):761–70). Statistical analyses were performed using SPSS version 26, employing the Chi-square test with a significance level set at p < 0.05.

**Results:**

The distribution of root canal morphologies varied significantly with age and gender. Among maxillary premolars, 50% exhibited the typical configuration of ^2^MPMB^1^ L^1^ (two roots, single canal in each buccal and lingual root), while 26% of maxillary right second premolars displayed ^1^MPM^1^ (one root, one canal). Overall, ^1^MPM^1^ accounted for 27.4% of the total cases in the second premolars. There was no statistically significant relationship between age and root canal distribution in either first premolars (p = 0.338) or second premolars (p = 0.833). Regarding gender, a significant difference was observed in the distribution of right maxillary 1st premolars (p = 0.022*), with a higher prevalence among females.

**Conclusion:**

This study offers significant insights into the anatomical variations of root canals in maxillary premolars across diverse regional subpopulations in Pakistan. While specific root canal configurations were prevalent, the findings indicate no statistically significant correlation between age and root canal morphology in maxillary premolars. However, a notable gender disparity was observed in the distribution of the right maxillary first premolars.

## Introduction

Root canal Configuration is a crucial determinant of root canal treatment success [2]. The differences in root canal morphologies present possibilities and difficulties in real endodontology [3, 4], and it remains the challenging aspect faced by an endodontist [5]. The complex system of root canal morphologies arises due to many diversities, such as accessory canals, isthmuses, and curvatures [6]. Overlooking these variations often results in inadequate root canal preparation and obturation, leading to endodontic failure and recurrent endodontic infections [7]. Hence, it becomes imperative that the clinician be cautious about variations in root canal configuration in the population; therefore, it can also be emphasized to utilize adequate and advanced imaging modalities to depict the root canal morphology precisely [8].

Root canal treatment conserves the tooth in its natural state and the surrounding periodontium [9]. A thorough evaluation and clinical workup are necessary to address the patient’s main complaint and utilize adequate and appropriate diagnostic instruments [10]. Moreover, possessing a thorough, comprehensive assessment of root canal configuration anatomy and the capability to interpret its structure accurately, coupled with proficiency in performing procedures, are crucial elements in achieving positive outcomes in endodontic therapy and minimizing post-treatment complications [11, 12].

Meticulous cleaning and shaping of the root canal ensure the success of the root canal treatment [13, 14]; however, the lack of awareness of various root canal morphology leads to inadequate biomechanical preparation and obturation. The complex nature of root canal morphology often leads to the failure of root canal treatments [15, 16]. Treating premolar teeth with root canal therapy has consistently presented difficulties for clinicians compared to other teeth [17, 18]. Various studies across diverse populations have frequently demonstrated the complex and varied root and canal structures in premolar teeth, often leading to new findings [19–21]. The significant diversity in the premolars has been highlighted in multiple studies, influenced by factors like ethnicity and geographic location [19–23].

Furthermore, to achieve results beneficial for endodontic therapy, it is essential to have a solid grasp of the variation in root and canal morphology [11]. However, changes in the anatomy of root canals, which include extra canals, curves, and isthmuses, might make the procedure utilizing instrumentation more difficult and increase the risk that the treatment will not be efficient overall [12, 24]. Hence, knowing the distribution of root canal configurations in a particular population might assist dentists in choosing suitable instrumentation techniques and improving the predictability of treatment [13].

Given the complex nature of root canal configurations, this study aimed to compile pertinent data on the root canal system of maxillary premolars in different subpopulations of Pakistan and understand the peculiar features using advanced imaging techniques. This will add to the current body of endodontic knowledge. Furthermore, by highlighting the therapeutic significance of our findings, we want to provide substantial insights for endodontic practitioners in this area.

## Materials and method

### Ethical consideration

Before commencing the study, ethical approval was obtained from the Aga Khan University Ethical Review Committee (Approval Number: 2024-1008-29149). This approval ensured that the study adhered to all ethical standards. The CBCT scans used in this research were initially acquired for therapeutic purposes unrelated to this study. To maintain patient confidentiality, strict protocols were implemented, including removing any personally identifiable information (PII) per widely accepted ethical guidelines.

### Sample selection

Two institutions contributed 707 CBCT scans to this retrospective cross-sectional study. The 592 CBCT images were procured from reputed dental clinics of Karachi’s Aga Khan University facility, signifying the pivotal role of the clinic in fostering groundbreaking research. A total of 115 scans were retrieved from the archives of Jinnah MRI and Body Scans in Lahore, Pakistan. The scanned images were then randomly selected using a simple random sampling technique for the study. Only scans that were taken for surgeries, orthodontic, endodontic therapy and implant design were considered. The inclusion criteria for a scan to be considered for analysis were for the tooth to exhibit normal radiological anatomy in its CBCT scans, such as well-formed roots and the absence of deep cavities. This stringent inclusion process ensured the data’s validity, reliability and quality of the dataset while reducing the probability of confounding variables and bias. Exclusion criteria involved teeth demonstrating gross decay, root canal calcifications, root anomalies, malformation, and full-coverage indirect prosthetic restorations. The methodology followed the study design guidelines of Martins et al. [14].

### Calibration

Prior to the investigation, a substantial cohort consisting of sixty CBCTs was selected to facilitate standardization in axial, sagittal and coronal sections by collaboration of two dental residents and an endodontist. Following this, a scoring criterion corroborated with the root canal morphology. A series of training sessions were conducted before the commencement of the study. During these sessions, the endodontist trained the other examiners to accurately interpret and classify the CBCT images according to Ahmed’s classification criteria. All examiners reviewed a total of 60 CBCT scans independently. In cases of disagreement, the images were re-evaluated collectively, and a consensus was reached through discussion. The rate of initial disagreement was approximately 10%, which decreased significantly as the examiners became more experienced. Throughout the calibration process, the three dental residents and the endodontist agreed with one another, as indicated by a Cohen kappa coefficient of 0.8.

### Analysis of root canal morphology

GALAXIS version 1.9 (SICAT GmbH and Co. KG, Bonn, Germany) was used to analyze CBCT images obtained from the Karachi centre. These images were displayed on a screen of 21 in. The data collection process began in the year 2020 and continued until the year 2023. Equipment manufactured by Sirona in Bensheim, Germany, was used to take the images presented here. The scanning was carried out at a voltage of 85 kV and a current magnitude of 7 milliamperes. The voxel size was 0.16 mm, and the exposure time was 15 s.

CBCTs retrieved from the scan centre were scans taken using Planmeca Promax 3D Classic (Planmeca, Helsinki, Finland), and visualization of the images was done using Planmeca Romexis version 6.4.3.33 (Planmeca Oy, Asentajankatu 6, FIN-00990, Helsinki, Finland) on a 21-in. screen monitor. The scanning was taken at 90 kV with 10 mA, with the voxel size of the image being 0.2 mm and an exposure time of 15 s. Based on the classification system by [1], the CBCts were examined in sagittal, coronal and axial sections by an endodontist with an experienced endodontist and two dental residents. Demographic data comprising age, gender and RCS data for each tooth were then coded in an Excel spreadsheet.

### Data analysis

The chi-square test was performed using the SPSS (Statistical Package for Social Services) software version 26 (IBM, Chicago, Illinois, USA). The test examined the prevalence of different root canal morphologies among distinct age groups and genders. The significance level was established at a p-value of less than 0.05. The Cohen kappa coefficient was used to calculate the agreements between examiners within the same examiner and between different examiners.

## Results

The demographic characteristics of the study population are summarized in Table 1. The age distribution showed that the most prominent groups were 21–30 years (22.2%) and 31–40 years (22.5%), with a mean age of 38.11 ± 14.98. Gender distribution was nearly balanced, with 47.2% males and 52.8% females. Regarding the type of teeth analyzed, right maxillary first premolars were the most common (26.38%), followed by left maxillary first premolars (25.60%), right maxillary second premolars (24.12%), and left maxillary second premolars (23.90%).

**Table 1:** Descriptive statistics of demographic characteristics

Variables	Frequency (n)	Percentage (%)
Age (n = 607)		
10–20	88	12.4
21–30	157	22.2
31–40	159	22.5
41–50	134	19.0
51–60	106	15.0
61–70	58	8.2
71–80	5	0.7
Gender		
Male	334	47.2
Female	373	52.8
Type of teeth (n = 2180)		
RM 1st PM	575	26.38
LM 1st PM	558	25.60
RM 2nd PM	526	24.12
LM 2nd PM	521	23.90

*RM* right maxillary, *LM* left maxillary, *PM* premolar

**Fig. 1:**
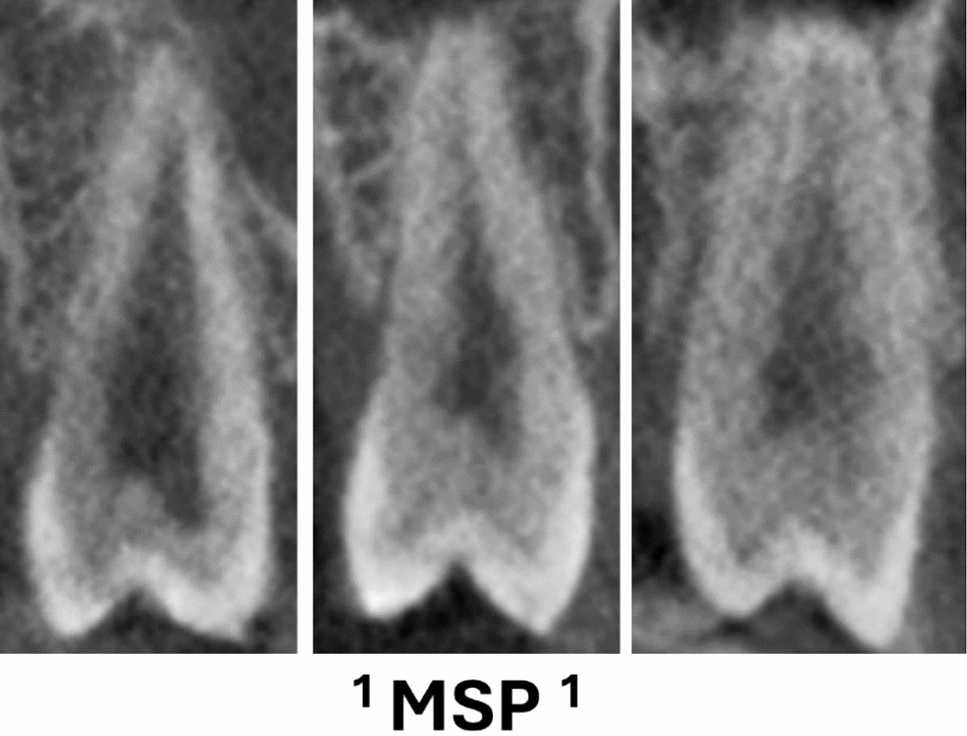
CBCT images (sagittal view) of maxillary second premolar (MSP) showing single code root canal morphology

**Fig. 2:**
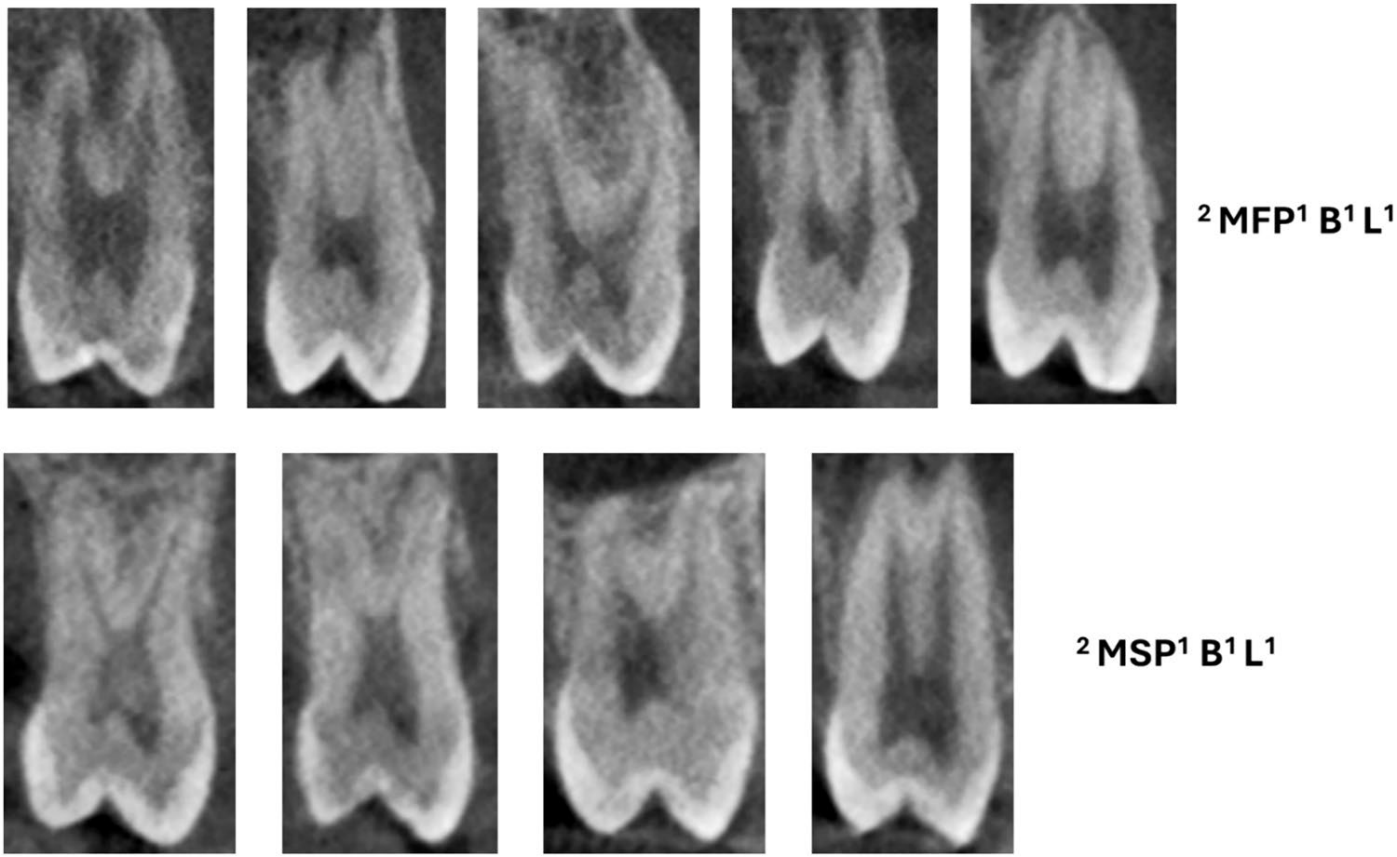
CBCT images (sagittal views) of maxillary premolar (MFP—first premolars, MSP—second premolars) showing root canal morphology with two roots

Table 2 displays the maxillary premolars distribution concerning the classification by [1], revealing varied configurations of root canals among the Pakistani subpopulations. Among the various classifications observed, the most common configuration for the maxillary right 1st premolar was ^2^MPM^1^ B^1^ L^1^, accounting for 50.1% of cases, and for the maxillary right 2nd premolar, ^1^MPM^1^ was common, comprising 26.0% of cases. Comparable patterns were also seen in the distribution on the left side, where ^1^MPM^1^ was the most common type in the second premolar (27.4%), and ^2^MPM^1^ B^1^ L^1^ was the most common in the first premolar (47.3%) (Figs. 1 and 2).

**Table 2:** Maxillary premolars distribution concerning to [1] classification

Codes	Max right 1st premolar n (%)	Max right 2nd premolar n (%)	Max left 1st premolar n (%)	Max left 2nd premolar n (%)
^1^MPM^1^	19 (3.3)	137 (26.0)	21 (3.8)	143 (27.4)
^1^MPM^2−1^	27 (4.7)	51 (9.7)	31 (5.6)	52 (10.0)
^1^MPM^1−2–1^	77 (13.4)	116 (22.1)	80 (14.3)	122 (23.4)
^1^MPM^2−2^	12 (2.1)	7 (1.3)	15 (2.7)	6 (1.2)
^1^MPM^1−2^	144 (19.8)	123 (23.4)	115 (20.6)	129 (24.8)
^1^MPM^2−1–2^	2 (0.3)	1 (0.2)	3 (0.6)	3 (0.6)
^1^MPM^1−2–1−2^	30 (5.2)	42 (8.0)	23 (4.1)	32 (6.1)
^1^MPM^2−1–2−1^	0	0	0	0
^2^MPMB^1^ L^1^	288 (50.1)	42 (8.0)	265 (47.5)	29 (5.6)
^1^MPM^1−3^	1 (0.2)	3 (0.6)	0	2 (0.4)
^1^MPM^1−3–1^	0	0	0	0
^1^MPM^1−3–2^	2 (0.3)	2 (0.4)	0	0
^1^MPM^1−2–3−2^	0	0	0	0
^1^MPM^2−3–2^	0	0	0	0
^1^MPM^1−2–3^	0	0	2 (0.4)	1 (0.2)
^1^MPM^1−2–4^	0	1 (0.2)	2 (0.4)	1 (0.2)
^1^MPM^1−2–1−3^	0	1 (0.2)	0	1 (0.2)
^3^MPM MB^1^DB^1^P^1^	3 (0.5)	0	1 (0.2)	0
Total	575	526	558	521

*MPM* maxillary premolar

**Fig. 3:**
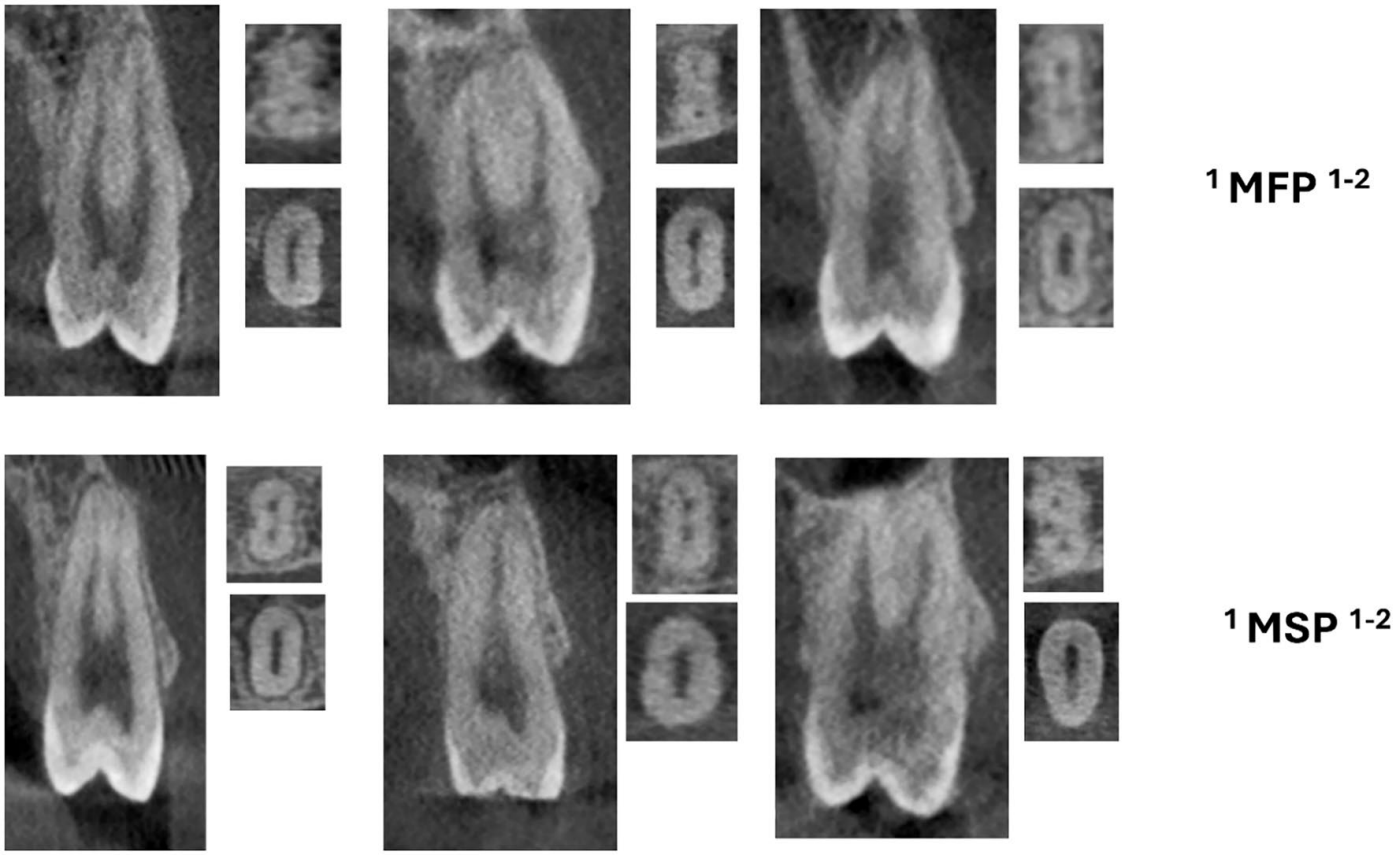
CBCT images (sagittal and axial views) of maxillary premolar (MFP—first premolars, MSP—second premolars) showing two code root canal morphology

Table 3 shows the distribution of maxillary right first premolars by age group. The total number of samples is 575, and their ages range from 11–20 to 71–80. The most prevalent classification across all age groups was ^2^MPM^1^ B^1^ L^1^, with the highest frequency observed in the 31–40 age range (65 cases). Across the same spectrum, ^1^MPM^1−2^ was consistently prevalent across all age groups, with the highest prevalence (29 occurrences) observed in the 21 to 30 age range (Fig. 3). A lack of statistically significant association between age and the distribution of right maxillary first premolars (p = 0.338) suggests that age plays little to no role in determining root canal designs in this particular cohort.

**Table 3:** Maxillary right 1st premolars distribution by age

MAX right 1st premolar	AGE								p-value
	11–20	21–30	31–40	41–50	51–60	61–70	71–80	Total	
^1^MPM^1^	3	5	5	3	1	2	0	19	0.338*
^1^MPM^2−1^	1	4	8	4	4	6	0	27	
^1^MPM^1−2–1^	6	23	8	14	19	7	0	77	
^1^MPM^2−2^	2	0	2	3	4	1	0	12	
^1^MPM^1−2^	10	29	21	26	17	9	2	114	
^1^MPM^2−1–2^	0	0	0	1	0	1	0	2	
^1^MPM^1−2–1−2^	4	7	11	3	4	1	0	30	
^2^MPM^1^ B^1^ L^1^	44	58	65	56	38	25	2	288	
^1^MPM^1−3^	1	0	0	0	0	0	0	1	
^1^MPM^1−2–3^	1	0	1	0	0	0	0	2	
^3^(MPM)^1^MB^1^DB^1^P^1^	0	1	0	1	1	0	0	3	
Total	72	127	121	111	88	52	4	575	

*MPM* maxillary premolar; Chi-square test

*Significant value < 0.05

Based on the age range, Table 4 displays the maxillary right second premolars distribution. While the ^1^MPM^1^ configuration was prevalent across all age groups, it was most common among those between the ages of 21 and 30 (33 cases). The most prevalent age group was those between 41 and 50 years old, with 29 cases, and ^1^MPM^1−2^ was consistently more common across various age categories. A p-value of 0.833 indicates that there is not a statistically significant association between the distribution pattern of the maxillary right 2nd premolars and the age of the participants.

**Table 4:** Maxillary right 2nd premolars distribution by age

MAX right 2nd premolar	AGE								p-value
	11–20	21–30	31–40	41–50	51–60	61–70	71–80	Total	
^1^MPM^1^	21	33	31	25	19	7	1	137	0.833
^1^MPM^2−1^	9	13	12	8	5	4	0	51	
^1^MPM^1−2–1^	17	25	22	22	17	12	1	116	
^1^MPM^2−2^	0	4	2	0	1	0	0	7	
^1^MPM^1−2^	14	29	21	29	17	13	0	123	
^1^MPM^2−1–2^	0	1	0	0	0	0	0	1	
^1^MPM^1−2–1−2^	4	9	9	10	5	4	1	42	
^2^MPM^1^ B^1^ L^1^	7	8	9	5	11	2	0	42	
^1^MPM^1−3^	0	0	0	1	1	1	0	3	
^1^MPM^1−2–3^	0	0	1	1	0	0	0	2	
^1^MPM^1−2–4^	0	0	0	0	0	1	0	1	
Total	72	122	107	101	77	44	3	526	

*MPM* maxillary premolar; Chi-square test

Table 5 shows the distribution of maxillary left first premolars by age group. Among all age categories, ^2^MPM^1^ B^1^ L^1^ stands out as the most common classification, with an enormous prevalence among those between the ages of 21 and 30 (66 cases). The highest occurrence was in the 41–50 age range (31 instances); however, ^1^MPM^1−2^ shows constant prevalence across different age categories. Regardless of these results, there was no statistically significant correlation between age and the distribution pattern of maxillary left first premolars (p = 0.519).

**Table 5:** Maxillary left 1st premolars distribution by age

MAX left 1st premolar	AGE								p-value
	11–20	21–30	31–40	41–50	51–60	61–70	71–80	Total	
^1^MPM^1^	3	5	4	3	4	2	0	21	0.519
^1^MPM^2−1^	3	6	12	3	5	2	0	31	
^1^MPM^1−2–1^	10	23	14	13	9	10	1	80	
^1^MPM^2−2^	3	1	5	4	2	0	0	15	
^1^MPM^1−2^	14	22	25	31	13	10	0	115	
^1^MPM^2−1–2^	0	0	0	1	1	1	0	3	
^1^MPM^1−2–1−2^	7	3	4	4	2	2	1	23	
^2^MPM^1^ B^1^ L^1^	32	66	56	51	40	17	2	264	
^1^MPM^1−2–3^	0	0	0	0	2	0	0	2	
^1^MPM^1−2–4^	0	0	1	0	1	0	0	2	
^3^(MPM)^1^MB^1^DB^1^P^1^	0	0	0	1	0	0	0	1	
Total	72	127	121	111	79	44	4	558	

*MPM* maxillary premolar; Chi-square test

Table 6 revealed that the data was broken down by age group for the maxillary left second premolars distribution. Notably, ^1^MPM^1^ is the most common category across all age groups, with the highest incidence observed in the 21–30 age range (34 cases). Similarly, whereas ^1^MPM^1−2^ is found in all age categories, it is consistently more common in the 31–40 age group (28 occurrences). No statistically significant relationship exists between age and the second premolars’ distribution pattern on the maxilla’s left side (p = 0.872).

**Table 6:** Maxillary left 2nd premolars distribution by age

MAX left 2nd premolar	Age								p-value
	11–20	21–30	31–40	41–50	51–60	61–70	71–80	Total	
^1^MPM^1^	23	34	29	26	19	11	1	143	0.872
^1^MPM^2−1^	5	16	11	4	11	5	0	52	
^1^MPM^1−2–1^	18	25	23	30	17	7	2	122	
^1^MPM^2−2^	0	2	2	0	2	0	0	6	
^1^MPM^1−2^	21	25	28	22	18	15	0	129	
^1^MPM^2−1–2^	1	2	0	0	0	0	0	3	
^1^MPM^1−2–1−2^	2	6	9	9	5	1	0	32	
^2^MPM^1^ B^1^ L^1^	1	8	5	6	4	4	0	28	
^1^MPM^1−3^	0	1	0	1	0	0	0	2	
^1^MPM^1−2–3^	0	0	0	1	0	0	0	1	
^1^MPM^1−2–4^	0	0	0	1	0	0	0	1	
Total	71	119	107	101	77	43	3	521	

*MPM* maxillary premolar; Chi-square test

The distribution of maxillary right first and second premolars, grouped by gender, is shown in Table 7. The sample consists of 575 maxillary right 1st premolars and 526 maxillary right 2nd premolars, including both males and females. The significance of the distribution concerning gender is evaluated using the p-value obtained from the Chi-square test. Notably, maxillary right 1st premolars demonstrate a statistically significant difference in distribution between males and females (p = 0.022*), with a higher prevalence observed among females. However, there appears to be a numerical difference in distribution between genders; the p-value (0.070) suggests no statistically significant association for maxillary right 2nd premolars in distribution between males and females across all classifications.

**Table 7:** Maxillary right 1st and 2nd premolars distribution by gender

MAX right 1st premolar	Gender			p-value	MAX right 2nd premolar	Gender			p-value
	Male	Female	Total			Male	Female	Total	
^1^MPM^1^	6	13	19	0.022*	^1^MPM^1^	72	65	137	0.070*
^1^MPM^2−1^	5	22	27		^1^MPM^2−1^	33	18	51	
^1^MPM^1−2–1^	34	43	77		^1^MPM^1−2–1^	45	71	116	
1MPM 2–2	3	9	12		^1^MPM^2−2^	2	5	7	
^1^MPM^1−2^	58	56	114		^1^MPM^1−2^	65	58	123	
^1^MPM^2−1–2^	1	1	2		^1^MPM^2−1–2^	0	1	1	
^1^MPM^1−2–1−2^	14	16	30		^1^MPM^1−2–1−2^	18	24	42	
2MPM^1^ B^1^ L^1^	153	135	288		^2^MPM^1^ B^1^ L^1^	20	22	42	
^1^MPM^1–3^	0	1	1		^1^MPM^1−3^	2	1	3	
^1^MPM^1−2–3^	0	2	2		^1^MPM^1−2–3^	2	0	2	
^3^(MPM)^1^MB^1^DB^1^P^1^	1	2	3		^1^MPM^1−2–4^	1	0	1	
Total	275	300	575		Total	261	265	526	

*MPM* maxillary premolar; Chi-square test

*Significant value < 0.05

**Fig. 4:**
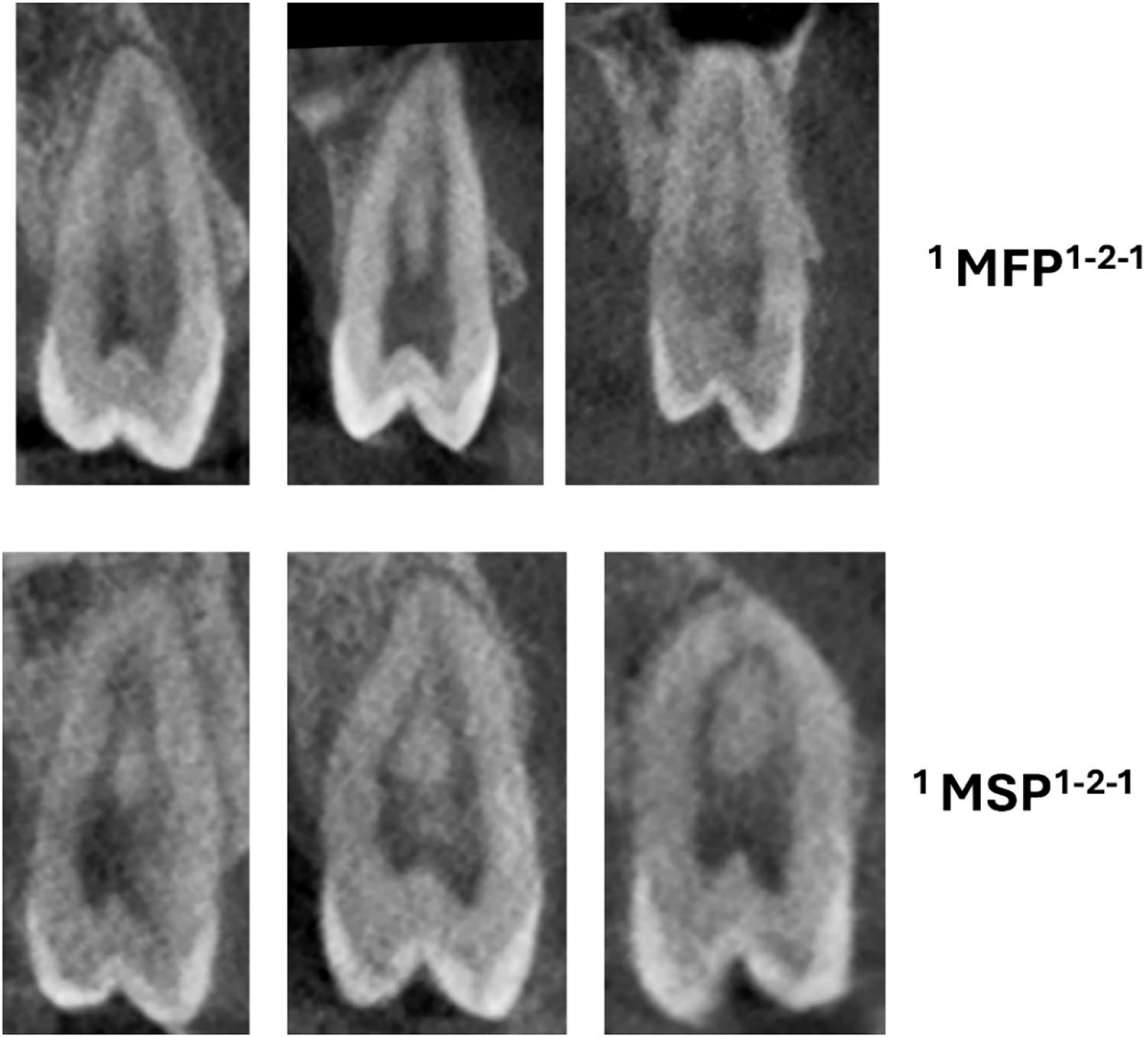
CBCT images (sagittal views) of maxillary premolar (MFP—first premolars, MSP—second premolars) showing three code root canal morphology

**Fig. 5:**
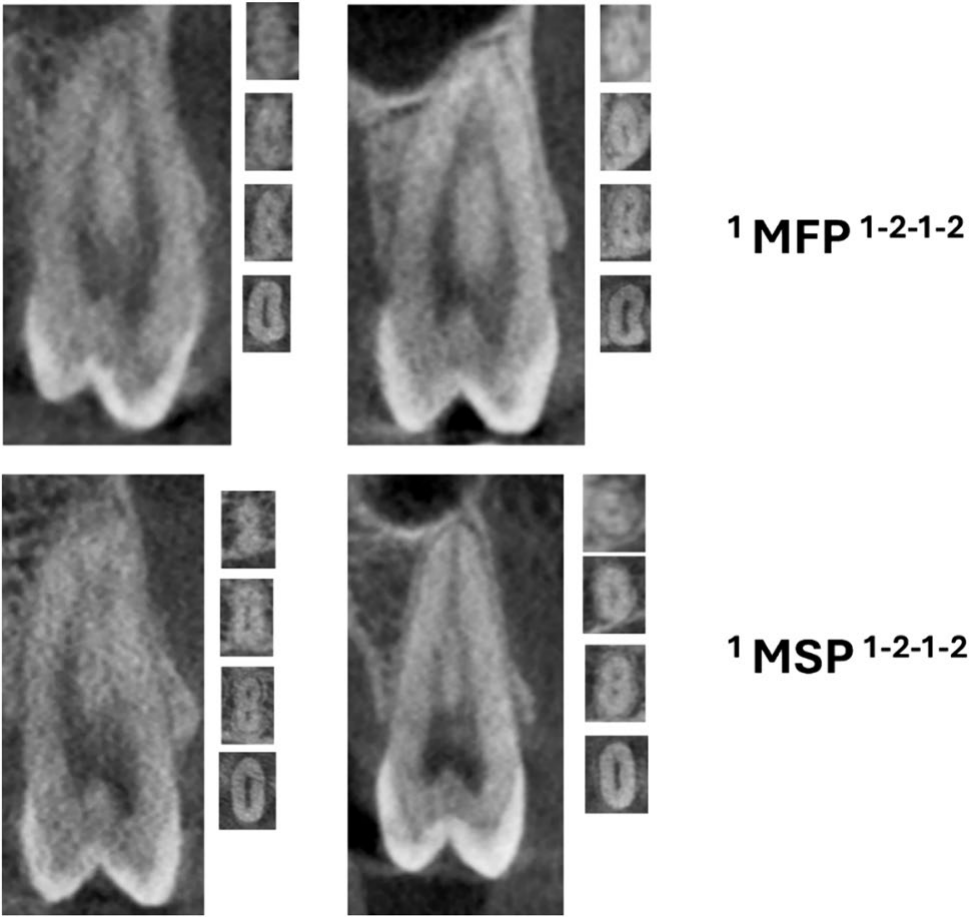
CBCT images (sagittal and axial views) of maxillary premolar (MFP—first premolars, MSP—second premolars) showing four code root canal morphology

Table 8 displays the maxillary left 1st and 2nd premolars distribution categorized by gender. The total sample size for maxillary left 1st premolars is 558, with 267 males and 291 females, while for maxillary left 2nd premolars, the total sample size is 521, with 259 males and 262 females. The statistical significance of the distribution concerning gender is assessed using the p-value obtained from the Chi-square test. Across all classifications for both maxillary left 1st and 2nd premolars, no statistically significant differences in distribution between males and females are observed (p > 0.05) (Figs. 4 and 5).

**Table 8:** Distribution of left maxillary 1st and 2nd premolars for gender

MAX left 1st premolar	Gender			p-value	MAX left 2nd premolar	Gender			p-value
	Male	Female	Total			Male	Female	Total	
^1^MPM^1^	11	10	21	0.575	^1^MPM^1^	67	76	143	0.355*
^1^MPM^2−1^	10	21	31		^1^MPM^2−1^	31	21	52	
^1^MPM^1−2–1^	37	43	80		^1^MPM^1−2–1^	64	58	122	
^1^MPM^2−2^	7	8	15		^1^MPM^2−2^	4	2	6	
^1^MPM^1−2^	61	54	115		^1^MPM^1−2^	62	67	129	
^1^MPM^2−1–2^	0	3	3		^1^MPM^2−1–2^	0	3	3	
^1^MPM^1−2–1−2^	10	13	23		^1^MPM^1−2–1−2^	12	20	32	
^2^MPM^1^ B^1^ L^1^	128	136	264		^2^MPM^1^ B^1^ L^1^	16	12	28	
^1^MPM^1−2–3^	1	1	2		^1^MPM^1−3^	1	1	2	
^1^MPM^1−2–4^	1	1	2		^1^MPM^1−2–3^	0	1	1	
^3^MPM MB^1^DB^1^P^1^	0	1	1		^1^MPM^1−2–4^	0	1	1	
Total	267	291	558		Total	259	262	521	

*MPM* maxillary premolar; Chi-square test

*Significant value < 0.05

## Discussion

Root canal treatment failure, particularly in premolars, can arise due to misdiagnosed canals caused by a lack of understanding of their complicated morphology [15]. Based on the findings of this study, CBCT emerges as the method of choice among the several techniques available for evaluating canal morphology. When imaging the root canal system, CBCT outperforms traditional radiography and displays accuracy on par with the staining-clearing approach [17]. Multiple studies show that extra canals can be more accurately identified with CBCT than conventional intra-oral radiography [19–21]. The use of CBCT in dentistry has resulted in revolutionary advancements in identifying and evaluating oral disorders [22]. Integrating CBCT imaging in the clinical setting enables the clinician to study dental anatomy in an improved, faster, less intrusive fashion and provides an accurate substitute compared to traditional in vitro analytical methods [25]. Various studies have used CBCT imaging to evaluate and study the root canal systems of all the teeth in human dentition, and significant revelations have been made, such as racial and ethnic variations among the root canal system being discovered [26, 27]. This study involving CBCT contributes to the growing body of literature by meticulously detailing the anatomical characteristics of maxillary incisors and premolars among the subpopulations in Pakistan.

The root canal morphology of maxillary premolars was categorized in our study using the [1] classification system, which played an integral part in precisely organizing the numerous root canal configurations observed in our datasets. Employing this classification system by [1] served as a benchmark and facilitated dependable assessment and precise documentation of diverse presentations of the root canal morphology. It also ensured reliability and validity in the results, enabling meaningful comparisons within the broader scope of endodontic research.

In our study, 50% of the root canals in our cohort were classified as having ^2^MPM^1^ B^1^ L^1^ canal system for the maxillary right first premolar, whereas 26% of the root canals in the maxillary right second premolar were classified as having ^1^MPM^1^. Similar findings were recorded in the left quadrant, wherein ^1^MPM^1^ was the most predominant pattern of root canal morphology in 27.4% of the second premolar, ^2^MPM^1^ B^1^ L^1^ system of the root canal was observed in 47.3% of the left first premolar. Prior studies have documented similar observations about the different morphologies of root canals in different populations [27–33]. These resonated with the findings of Nazeer et al. [25], who observed subpopulations and provided substantial evidence for the prevalence of these morphologies in maxillary premolars in Pakistanis. Celikten et al. [34] and Tian et al. [35] reported a higher prevalence of maxillary premolars with a single root. Environmental and genetic factors influence the diversity in root canal morphologies observed among various ethnic groups.

Further, the research conducted by Parolia et al. [36] employed CBCT imaging to investigate the root and root canal morphology of maxillary premolars. The study aimed to analyze the influence of demographic factors on the results. A worldwide evaluation of maxillary premolars’ root and root canal features has identified a noticeable influence of different factors, including tooth type, geographical region, ethnicity, sex, and age. Furthermore, compared to the findings of a recent systematic review with meta-analysis by Martins et al. [14], which performed a quality assessment of in vivo studies using CBCT imaging to evaluate root canal morphology, our results align with global trends reported in their study. Martins et al. emphasized that geographic region, sample size, and CBCT settings significantly impact the quality and findings of such studies. Although limited to a Pakistani subpopulation, our study adheres to high methodological standards by utilizing CBCT imaging and a comprehensive classification system.

In Maxillary right and left premolars, ^2^MPM^1^ B^1^ L^1^ configuration emerged consistently as the frequent pattern amongst all the age groups. Albeit the predominant pattern, no statistically significant correlation was observed between the age and the pattern of RCS distribution between the maxillary left and right premolars. Our observations in the context of specific root canal configurations, such as ^1^MPM^1−2^ and ^2^MPM^1^ B^1^ L^1^, were concurrent with the literature findings where similar investigations have been conducted with a different geographic [37]. From the findings mentioned above, it is evident that age alone cannot be the determining factor for the plethora of root canal morphologies within our particular subpopulation. The absence of substantial changes in root canal morphology distribution concerning age during our research could be attributed to several factors. One is the statistical power, which detects that the differences can be influenced by the size and effect size of the sample population. A lower or inadequate sample size often lacks statistical power to differentiate minor variations among distinct age cohorts [38]. Similar patterns of root canal morphologies across diverse age groups lead to decreased chances of obtaining a significant difference. This scenario is more likely when the population under study is homogenous and shares similar genetic or cultural characteristics [39, 40]. Furthermore, when age groups are broadly defined or overlap, it poses significant challenges in detecting prospective alterations in root canal morphologies that occur with ageing [41]. The lack of substantial differences could be extended to the inherent variability in root canal morphology, which varies not only by age but also due to environmental factors, genetics, and dental interventions [42].

Our study confirms and validates the findings of Ketabi et al. [43], who described the existence of three separate root canals in three different maxillary first premolars. They also stated that around 1.85% of their cohort’s maxillary first premolars exhibited three roots. The findings documented in the literature and studies that employed CBCT scan as an imaging modality to study RCS resonated with our findings. Commonly, these levels fall between 0 and 6% [25, 29, 33–35, 44, 45].

Based on our findings, the predominant patterns of root canal morphologies of maxillary premolars in both quadrants within our cohort closely mirror the findings reported in prior research works with minor alterations, albeit not significant. Neelakantan et al. [40] and Asheghi et al. [46 found similar rates of prevalence for prevalence rates were observed for distinct root canal morphologies like ^1^MPM^1−2^ and ^1^MPM^1^. This indicates that root canal morphology might remain consistent across different age groups. Nevertheless, methodological differences, sample size and demographic factors could result in discrepancies. The findings of Ketabi et al. [43] and observations from this study raise the prospect of regional or ethnic differences that alter root canal shape. The observations also agreed with Bulut et al. [47] and Garg et al. [42], who demonstrated no substantial correlation between age and the location of root canals. Further investigation is necessary to identify the elements that affect the shape of root canals, in addition to age, as suggested by the findings of this study.

Gender is one of the potential additional factors that can affect the number of roots and the shape of the roots [27, 29, 48]. To determine the degree of significance of the gender distribution, we utilized the p-value derived from the Chi-square test. It is important to note a significant difference in the maxillary right first premolars distribution between males and females, with a higher prevalence reported in females (p = 0.022*). This is something that should be taken into consideration. This substantiates the findings of earlier studies, which state that the form of the root canal differs between the sexes [49–51]. Surprisingly, Ahmad et al. 52 revealed that root canal morphology differed significantly across both genders and that men had a significantly greater number of canals than women. The lack of the categorization code ^1^MPM^1–2–1^ in Ahmed et al.’s research hinders direct comparisons. Mashyakhy [53] found significant root canal morphological differences between Saudi Arabian males and females in maxillary teeth. Martins et al. [54] found gender differences in the root canal morphology of Portuguese populations. Different subgroups got different outcomes. Dentists should recognize these discrepancies and use their abilities and devices to treat complex canal structures. Root canal identification and navigation instructions and tools are available in the literature. Detailed radiograph analysis and interpretation, creating a suitable access cavity, thoroughly evaluating the pulp chamber floor to identify canal openings, and using magnifying instruments like dental loupes to improve visibility during treatment [55, 56].

Regarding the classification of right maxillary 2nd premolars, there appears to be a difference in the number of occurrences between males and females. However, the p-value (0.070) indicates no statistically significant relationship. Comparable results were noted in research conducted on subgroups from Malaysia [57] and Germany [58]. Based on these results, gender might influence the prevalence of specific root canal designs in maxillary first premolars, but it might not significantly affect the distribution pattern of maxillary second premolars across different groups. As a result of the vast geographical breadth of our nation, it is possible that the findings of our research will not apply to all subsets of the Pakistani population. Therefore, it is recommended that future studies examine the number of roots and the shape of canals in tooth groups that are similar and originate from different demographic locations. Further investigations are warranted to explore the correlation of gender, tooth location and dental anatomy in determining the root canal morphology.

### Clinical implications

The findings of this study can be extrapolated to clinical settings for several reasons. Firstly, the success of root canal treatment depends on a thorough understanding of the diverse root canal anatomy in the maxillary premolars of Pakistanis. This would allow the clinicians and endodontists to sharpen their skills in negotiating complex root canal morphologies. Secondly, the discrepancies in root canal anatomy between genders emphasize the need for patient-specific treatment approaches. Dentists should consider these gender-specific variations when planning for a root canal treatment to achieve an optimal success rate. Moreover, the variations in root canal morphology associated with age also highlight the importance of customized treatment protocols, particularly for elderly individuals exhibiting diverse canal configurations, for the long-term success of root canal treatment.

### Limitations

The limitations of our study need to be addressed. Although our dataset was large, the sample size might not fully represent the entire Pakistani population due to its retrospective nature and potential selection bias. The accuracy of the results could be affected by the exclusion criteria and differences in the interpretation of the CBCT images. Moreover, relying on a single classification system could have overlooked some morphological variations. The study’s cross-sectional design also limits the ability to establish causal relationships. Future research should aim to overcome these constraints by incorporating more diverse and extensive datasets, utilizing multiple classification schemes, and adopting longitudinal study designs to understand the complexities of root canal morphology better. Additionally, potential biases such as selection bias and observer bias should be minimized in future studies.

## Conclusion

This study offers significant insights into the anatomical variations of root canals in maxillary premolars across diverse regional subpopulations in Pakistan. While specific root canal configurations were prevalent, the findings indicate no statistically significant correlation between age and root canal morphology in maxillary premolars. However, a notable gender disparity was observed in the distribution of the right maxillary first premolars.

## Data Availability

All data supporting the findings of this study are available from the corresponding author upon reasonable request.

## References

[1] Ahmed H, Versiani M, De-Deus G, et al. A new system for classifying root and root canal morphology. Int Endod J. 2017;50(8):761–70. 10.1111/iej.12685 27578418

[2] Ahmed HMA. A critical analysis of laboratory and clinical research methods to study root and canal anatomy. Int Endod J. 2022;55:229–80. 35124829 10.1111/iej.13702

[3] Karobari MI, et al. Root and root canal morphology classification systems. Int J Dent. 2021;2021:1–6. 10.1155/2021/6682189 PMC7910041 33679981

[4] Karobari MI, et al. Root and root canal configuration characterization using microcomputed tomography: a systematic review. J Clin Med. 2022;11(9):2287. 35566414 10.3390/jcm11092287 PMC9099997

[5] Ahmed HMA, et al. Application of a new system for classifying root and canal anatomy in clinical practice—explanation and elaboration. Eur Endod J. 2021;6(13):132–42. 34650010 10.14744/eej.2021.38257 PMC8461493

[6] Versiani M, et al. Anatomical complexities affecting root canal preparation: a narrative review. Aust Dent J. 2023;68:S5–23. 37984802 10.1111/adj.12992

[7] Mustafa M, et al. Evaluation of the causes of failure of root canal treatment among patients in the City of Al-Kharj, Saudi Arabia. Niger J Clin Pract. 2021;24(4):621–8. 33851687 10.4103/njcp.njcp_290_20

[8] Maghfuri S, et al. Evaluation of root canal morphology of maxillary first premolars by cone beam computed tomography in Saudi Arabian southern region subpopulation: an in vitro study. Int J Dent. 2019;15(12):3663. 10.1155/2019/2063943 PMC6415280 30936918

[9] Javed J, et al. Evaluation of undergraduate dental students self-perceived confidence level regarding endodontic procedures: a questionnaire survey. Saudi Endod J. 2021;11(2):228–34. 10.4103/sej.sej_155_20

[10] Hanif F, et al. Frequency of root canal configurations of maxillary premolars as assessed by cone-beam computerized tomography scans in the Pakistani subpopulation. Saudi Endod J. 2022;12(1):100–5. 10.4103/sej.sej_141_21

[11] Mustafa M, et al. Assessment of the root and canal morphology in the permanent dentition of Saudi Arabian population using cone beam computed and micro-computed tomography—a systematic review. BMC Oral Health. 2024;24(1):343. 38493123 10.1186/s12903-024-04101-3 PMC10944621

[12] ChuppaniDastgerdi A, et al. Isthmuses, accessory canals, and the direction of root curvature in permanent mandibular first molars: an in vivo computed tomography study. Restor Dent Endod. 2020;45(1): e7. 32110536 10.5395/rde.2020.45.e7 PMC7030962

[13] Kirilova J, Topalova-Pirinska S. C-shaped configuration of the root canal system—problems and solutions. J Int Med Assoc Belarus Annu Proc (Sci Pap). 2014;20:504–9.

[14] Martins JN, et al. Preferred reporting items for epidemiologic cross-sectional studies on root and root canal anatomy using cone-beam computed tomographic technology: a systematized assessment. J Endod. 2020;46(7):915–35. 32387077 10.1016/j.joen.2020.03.020

[15] Iqbal A. The factors responsible for endodontic treatment failure in the permanent dentitions of the patients reported to the college of dentistry, the University of Aljouf, Kingdom of Saudi Arabia. J Clin Diagn Res. 2016;10(5):ZC146. 27437351 10.7860/JCDR/2016/14272.7884 PMC4948527

[16] Karobari MI, et al. Application of two systems to classify the root and canal morphology in the human dentition: a national survey in India. J Dent Educ. 2023;87(8):1089–98. 37164913 10.1002/jdd.13236

[17] Neelakantan P, et al. Comparative evaluation of modified canal staining and clearing technique, cone-beam computed tomography, peripheral quantitative computed tomography, spiral computed tomography, and plain and contrast medium—enhanced digital radiography in studying root canal morphology. J Endod. 2010;36(9):1547–51. 20728725 10.1016/j.joen.2010.05.008

[18] Karobari MI, et al. Root and canal morphology of the anterior permanent dentition in Malaysian population using two classification systems: a CBCT clinical study. Aust Endod J. 2021;47(2):202–16. 33159714 10.1111/aej.12454

[19] Issac E. A comparative study to evaluate the accuracy of CBCT, digital radiography and intra oral periapical radiography for the assessment of the anatomy of the maxillary second premolar root canals: an in vitro study. Bengaluru: Rajiv Gandhi University of Health Sciences; 2019.

[20] Singh N, et al. Comparative analysis of the accuracy of periapical radiography and cone-beam computed tomography for diagnosing complex endodontic pathoses using a gold standard reference—a prospective clinical study. Int Endod J. 2021. 10.1111/iej.13535. 33904603 10.1111/iej.13535

[21] McGuigan M. Aspects of dose optimisation and diagnostic efficacy relating to dental cone beam computed tomography. Dublin: Trinity College Dublin; 2023. 10.61872/sdj-2018-04-395 29589667

[22] Ahluwalia R, et al. The use of CBCT in dentistry represents the future of the profession. J Surv Fish Sci. 2023;10:1088–93.

[23] Karobari MI, et al. Roots and root canals characterization of permanent mandibular premolars analyzed using the cone beam and micro computed tomography—a systematic review and metanalysis. J Clin Med. 2023;12(6):2183. 36983187 10.3390/jcm12062183 PMC10051908

[24] Karobari MI, et al. Evaluation of root and canal morphology of mandibular premolar amongst Saudi subpopulation using the new system of classification: a CBCT study. BMC Oral Health. 2023;23(1):291. 37189077 10.1186/s12903-023-03002-1 PMC10184334

[25] Nazeer MR, et al. Evaluation of root morphology and canal configuration of maxillary premolars in a sample of Pakistani population by using cone beam computed tomography. J College Physicians Surg-Pak. 2018;68(3):423. 29540878

[26] Almansour MI, et al. Comprehensive evaluation of root and root canal morphology of mandibular second molars in a Saudi subpopulation evaluated by cone-beam computed tomography. BMC Oral Health. 2022;22(1):267. 35778729 10.1186/s12903-022-02305-z PMC9250273

[27] Martins JN, et al. Differences on the root and root canal morphologies between Asian and white ethnic groups analyzed by cone-beam computed tomography. J Endod. 2018;44(7):1096–104. 29861062 10.1016/j.joen.2018.04.001

[28] Kartal N, et al. Root canal morphology of maxillary premolars. J Endod. 1998;24(6):417–9. 9693586 10.1016/S0099-2399(98)80024-1

[29] Abella F, et al. Cone-beam computed tomography analysis of the root canal morphology of maxillary first and second premolars in a Spanish population. J Endod. 2015;41(8):1241–7. 25956606 10.1016/j.joen.2015.03.026

[30] Loh H. Root morphology of the maxillary first premolar in Singaporeans. Aust Dent J. 1998;43(6):399–402. 9973709 10.1111/j.1834-7819.1998.tb00199.x

[31] Senan EM, et al. Root form and canal morphology of maxillary first premolars of a Yemeni population. BMC Oral Health. 2018;18:1–10. 29855300 10.1186/s12903-018-0555-x PMC5984329

[32] Yang L, et al. Use of cone-beam computed tomography to evaluate root canal morphology and locate root canal orifices of maxillary second premolars in a Chinese subpopulation. J Endod. 2014;40(5):630–4. 24767555 10.1016/j.joen.2014.01.007

[33] Alqedairi A, et al. Cone-beam computed tomographic evaluation of root canal morphology of maxillary premolars in a Saudi population. BioMed Res Int. 2018;2018:8170620. 30186867 10.1155/2018/8170620 PMC6114071

[34] Celikten B, et al. Cone-beam CT evaluation of root canal morphology of maxillary and mandibular premolars in a Turkish Cypriot population. BDJ Open. 2016;2(1):1–5. 10.1038/bdjopen.2015.6 PMC5831013 29607060

[35] Tian YY, et al. Root and canal morphology of maxillary first premolars in a Chinese subpopulation evaluated using cone-beam computed tomography. Int Endod J. 2012;45(11):996–1003. 22551454 10.1111/j.1365-2591.2012.02059.x

[36] Parolia A, et al. Worldwide assessment of the root and root canal characteristics of maxillary premolars—a multi-center CBCT cross-sectional study with meta-analysis. J Endod. 2024;50(1):31–54. 37898333 10.1016/j.joen.2023.10.009

[37] Ahmed H, et al. Application of a new system for classifying root canal morphology in undergraduate teaching and clinical practice: a national survey in Malaysia. Int Endod J. 2020;53(6):871–9. 32003029 10.1111/iej.13271

[38] Malterud K, et al. Information power: sample content and size in qualitative studies. 2021.

[39] Gulabivala K, et al. Root and canal morphology of Burmese mandibular molars. Int Endod J. 2001;34(5):359–70. 11482719 10.1046/j.1365-2591.2001.00399.x

[40] Neelakantan P, et al. Cone-beam computed tomography study of root and canal morphology of maxillary first and second molars in an Indian population. J Endod. 2010;36(10):1622–7. 20850665 10.1016/j.joen.2010.07.006

[41] Cleghorn BM, et al. The root and root canal morphology of the human mandibular second premolar: a literature review. J Endod. 2007;33(9):1031–7. 17931927 10.1016/j.joen.2007.03.020

[42] Garg N, et al. Mandibular premolars with bifurcated canals. Dental J Adv Stud. 2020;8(01):32–4. 10.1055/s-0040-1709096

[43] Ketabi M, et al. Evaluation of root morphology of maxillary first premolars. J Isfahan Dental School. 2008;4(3):162–9.

[44] Popović M, et al. Cone-beam computed tomography study of root number and root canal configuration of premolars in Serbian population. Med Pregl. 2018;71(3–4):100–7. 10.2298/MPNS1804100P

[45] Ok E, et al. A cone-beam computed tomography study of root canal morphology of maxillary and mandibular premolars in a Turkish population. Acta Odontol Scand. 2014;72(8):701–6. 24832561 10.3109/00016357.2014.898091

[46] Asheghi B, Momtahan N, Sahebi S, Zangoie Booshehri M. Morphological evaluation of maxillary premolar canals in Iranian population: A cone-beam computed tomography study. J Dent. 2020;21(3):215–24. 10.30476/DENTJODS.2020.82299.1011 PMC7519931 33062816

[47] Bulut DG, Kose E, Ozcan G, et al. Evaluation of root morphology and root canal configuration of premolars in the Turkish individuals using cone beam computed tomography. Eur J Dent. 2015;9(4):551–7. 10.4103/1305-7456.172624 PMC4745238 26929695

[48] Sert S, Bayirli GS. Evaluation of the root canal configurations of the mandibular and maxillary permanent teeth by gender in the Turkish population. J Endod. 2004;30(6):391–8. 15167464 10.1097/00004770-200406000-00004

[49] Karobari MI, et al. Assessment of root canal morphology of maxillary premolars: a CBCT study exploring age and gender variations. 2023. 10.1186/s12903-024-04310-w PMC11084092 38724952

[50] Syed GA, et al. CBCT evaluation of root canal morphology of maxillary first premolar in Saudi subpopulation. J Pharm Bioallied Sci. 2024;16(Suppl 2):S1619–22. 38882762 10.4103/jpbs.jpbs_1048_23 PMC11174182

[51] Lemos MC, et al. Root canal morphology of 1316 premolars from Brazilian individuals: an in vivo analysis using cone-beam computed tomography. Acta Odontol Latinoam. 2022;35(2):105–10. 36260941 10.54589/aol.35/2/105 PMC10283387

[52] Ahmad IA, Alenezi MA. Root and root canal morphology of maxillary first premolars: A literature review and clinical considerations. J Endod. 2016;42(6):861–72. 10.1016/j.joen.2016.02.017 27106718

[53] Mashyakhy M. Anatomical evaluation of maxillary premolars in a Saudi population: an in vivo cone-beam computed tomography study. J Contemp Dent Pract. 2021;22(3):284–9. 34210930 10.5005/jp-journals-10024-3070

[54] Martins JN, et al. Gender influence on the number of roots and root canal system configuration in human permanent teeth of a Portuguese subpopulation. Quintessence Int. 2018;49(2):103–11. 29234740 10.3290/j.qi.a39508

[55] Cantatore G, et al. Missed anatomy: frequency and clinical impact. Endod Top. 2006;15(1):3–31. 10.1111/j.1601-1546.2009.00240.x

[56] Vertucci FJ. Root canal morphology and its relationship to endodontic procedures. Endod Top. 2005;10(1):3–29. 10.1111/j.1601-1546.2005.00129.x

[57] Pan JYY, et al. Root canal morphology of permanent teeth in a Malaysian subpopulation using cone-beam computed tomography. BMC Oral Health. 2019;19:1–15. 30642318 10.1186/s12903-019-0710-z PMC6332542

[58] Bürklein S, et al. Evaluation of the root canal anatomy of maxillary and mandibular premolars in a selected German population using cone-beam computed tomographic data. J Endod. 2017;43(9):1448–52. 28743430 10.1016/j.joen.2017.03.044

